# Effect of *Coridothymus capitatus* Essential Oil on Chrysanthemum Aphid Behaviour and Survival: Phytochemical Analysis and Antioxidant Potential

**DOI:** 10.3390/molecules30163437

**Published:** 2025-08-20

**Authors:** Paraskevi Yfanti, Andreas Papavlasopoulos, Polyxeni Lazaridou, Dimitra Douma, Marilena E. Lekka

**Affiliations:** 1 Department of Agriculture, University of Ioannina, 47150 Arta, Greece; 2 Independent Researcher, 45332 Ioannina, Greece; andreas_papavlasopoulos@yahoo.com (A.P.); dimitra@ddoumasons.com (D.D.); 3 Department of Chemistry, University of Ioannina, 45110 Ioannina, Greece; p.lazaridou@uoi.gr

**Keywords:** *Thymus capitatus*, *Thymbra capitata*, *Satureja capitata*, essential oil, *Macrosiphoniella sanborni*, DPPH

## Abstract

There is a growing interest in using essential oils with phytoprotectant properties instead of synthetic pesticides to mitigate the risks of insect pesticide resistance, environmental harm, and adverse effects on non-target organisms and human health. This study focused on the effects of *Coridothymus capitatus* essential oil on host selection, settling behaviour, and survival of *Macrosiphoniella sanborni* in dual-choice and no-choice tests. The essential oil and methanol extract of *C. capitatus* were analyzed using Gas Chromatography–Mass Spectrometry (GC-MS) and Liquid Chromatography–Mass Spectrometry (LTQ-LC-MS Orbitrap), respectively. The antioxidant activity was also tested through the radical scavenging assay. The settling inhibitory activity in the dual-choice test increased dose-dependently from 60% to 72% for essential oil concentrations of 0.1 to 0.3% (*v*/*v*) for up to 120 min exposure, but decreased thereafter. However, under no-choice conditions, the inhibitory effect after 60 min of exposure was inversely proportional to the concentration but became proportional by the end of the experiment (72 h). After 72 h, both assays produced a mortality rate of 15% to 17%. *C. capitatus* was classified as a Carvacrol chemotype. Fifteen phenolic compounds were identified in the MeOH extract, and both the extract and essential oil exhibited substantial antioxidant activity. In conclusion, our findings indicate that *C. capitatus* essential oil affects the behaviour and survival of *M. sanborni*.

## 1. Introduction

The excessive use of chemical pesticides has led to significant environmental issues, including soil and groundwater pollution, which threaten both beneficial and non-target organisms and endanger human health. Additionally, their overuse contributes to the development of chemoresistance in pest populations, leading to the need for increasing concentrations of chemicals to achieve the same level of control. These challenges have highlighted the urgent need for safer, more sustainable alternatives in plant protection. In response, attention has turned to natural biodegradable compounds derived from plants. Among these, plant essential oils—classified as secondary metabolites with allelochemical properties [1]—have gained prominence. Their biodegradability, minimal toxicity to non-target organisms, reduced likelihood of resistance development, and multiple modes of action position them as a promising and eco-friendly alternative to conventional chemical pesticides [2].

The ecological role of these compounds is crucial for the establishment and spread of plants in their environment. Several ingredients of essential oils may serve as attractants for beneficial organisms (pollinators and natural enemies of pests) [3] or exert protective activity, as part of the plant’s chemical defence system, against phytopathogens and herbivorous organisms [4]. Some organisms have developed the ability to metabolize these compounds, enabling them to effectively attack plants. Essential oils also exhibit a selective impact depending on their chemical composition and the targeted organism, making them a viable option for agricultural pest management in organic farming. Despite their encouraging insecticidal and repellent characteristics, the practical deployment of plant essential oils often comes with a number of potential issues and drawbacks, such as volatility and short residual activity, formulation challenges [5], and phytotoxicity [6].

Field and glasshouse crops are significantly affected by aphids (Homoptera: Aphididae), which are considered serious pests. Aphid morphs include alatae and apterous individuals that are mostly reproduced asexually. Aphids negatively affect plants by piercing plant tissues and extracting phloem sap. Moreover, they can serve as vectors for numerous viral diseases. Extensive infestations can result in the production of honeydew, which attracts ants and contributes to the growth of black, sooty mould that inhibits photosynthesis [7].

To avoid aphid infestations, plants use different methods of defence, both direct and indirect. As a part of their direct defence strategy to impede aphid growth and development, they can produce a combination of chemical compounds, such as flavonoid and phenolic compounds, tannins, latex, and extrafloral nectar. Under natural conditions, aphid populations can increase rapidly. Despite the implementation of intense control measures, aphid species have expanded into new areas and inflicted damage on crops worldwide. On average, aphid damage reduces yields by approximately 30% to 50% each year [8,9,10].

Chrysanthemum (*Chrysanthemum morifolium* Ramat.) is a highly prized plant that is vulnerable to aphids (Chrysanthemum aphid—*M. sanborni*), which can cause substantial damage. Chrysanthemum is widely grown for its ornamental value and medicinal properties, which is why it is used for infusion in tea preparations [11,12,13]. *M. sanborni* has a detrimental effect on both plant growth and flower quality, leading to substantial losses in chrysanthemum production. In addition to depleting plants of nutrients, aphids can carry pathogenic viruses [14,15]. Consequently, it is essential to develop an efficient and eco-conscious control system for the chrysanthemum aphid.

*Coridothymus capitatus* (L), also known as conehead thyme or Spanish oregano, is a perennial herbaceous shrub from the Lamiaceae family. Other species synonyms with *C. capitatus* are *Thymbra capitata* (L.) Cav., *Satureja capitata* (L.), and *Thymus capitatus* (L.) Hoffmanns and Link. This species has been traditionally used in the Mediterranean region for its medicinal properties [16] and as a culinary herb for food aromatization and improvement of organoleptic properties [17]. Essential oil, with its antibacterial and antioxidant properties, is ideal for use as a natural preservative in the food industry. Essential oils [18] or other secondary plant metabolites also obtained via crop residues [19], with the above properties, can also be used as additives in natural biopolymer films for the formation of bioactive and biodegradable food packaging to extend the shelf life of perishable fruits. Due to the anti-inflammatory and anti-aging activities of the essential oil, they are widely used in the cosmetics industry [20]. Researchers have also focused on the antifungal [21,22] and antibacterial [23,24] properties of *C. capitatus* essential oil, against human or animal pathogens, foodborne pathogens, and phytopathogens. However, less attention has been paid to its antiviral and insecticidal activity [25,26]. The fumigant activity of essential oils has been extensively studied in relation to their insecticidal effect on insects that affect stored products [3].

This study aimed to evaluate the effect of *C. capitatus* essential oil on the settling behaviour and survival of the crysanthemum aphid *M. sanborni*, considering the chemical composition of the essential oil and methanol extract obtained from a wild population of this aromatic herb in Epirus, Greece. The free radical scavenging activity of the essential oil was assessed.

## 2. Results

### 2.1. Effect of Essential Oil on Aphid Behaviour and Survival

**Figure 1:**
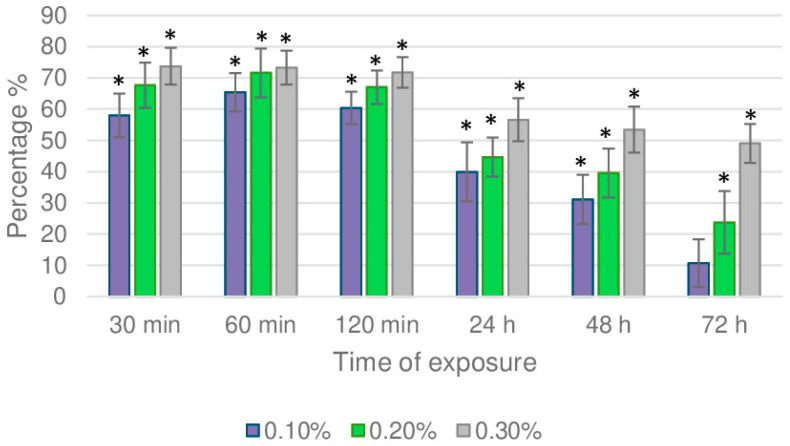
Chrysanthemum aphid settling inhibition index (SI%) on chrysanthemum leaf discs treated with *C. capitatus* essential oil, as determined in a 72 h dual-choice bioassay. Ten adult aphids were applied per treatment. Results are expressed as mean values and standard errors for 40 biological replicates. Asterisks indicate statistically significant differences in aphid numbers on treated and control-untreated chrysanthemum leaf discs (Triton X-100, 0.5%) at *p* ≤ 0.05 (Wilcoxon’s matched pair signed rank test).

The essential oil inhibited aphids from settling on the treated leaf discs both in dual-choice and no-choice experiments. The settling inhibitory activity observed by the dual-choice test (Figure 1) was dose-dependent and reached 58 to 74% after 30 min (*p* = 0.000, Wilcoxon’s test). It is worth mentioning that the inhibitory activity decreased as the exposure time increased. By the end of the experiment (after 72 h), this percentage was significantly lower, reaching a reduction rate of up to 23% and 46% for 0.1% and 0.3% concentrations, respectively (*p* = 0.005, *p* = 0.000, Wilcoxon’s test).

**Figure 2:**
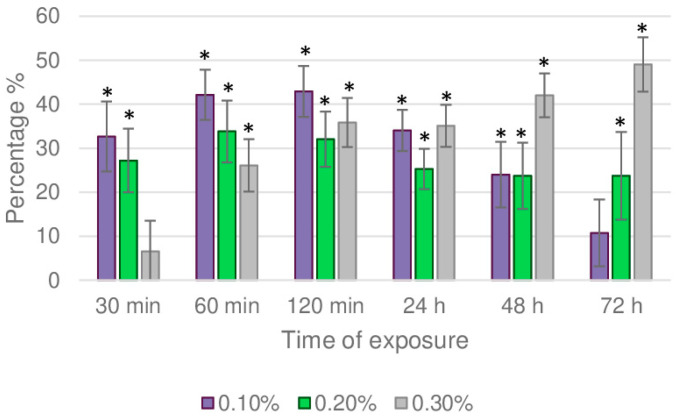
Chrysanthemum aphid settling inhibition index (SI%) on chrysanthemum leaf discs treated with *C. capitatus* essential oil, as determined in a 72 h no-choice test. Results are expressed as mean values and standard errors for 20 biological replicates, with 10 adult aphids per treatment. Asterisks indicate statistically significant difference between the number of aphids settled on the treated or the control leaf discs (Triton X-100, 0.5%) at *p* ≤ 0.05 (Kruskal–Wallis Test).

At the beginning of the time- and dose-dependent experiment (30 min) under no-choice conditions, the settling inhibitory activity (Figure 2) was significantly higher (33%) for the 0.1% concentration (*p* = 0.001, Kruskal–Wallis), while at the highest concentration (0.3%), the difference was not significant, (*p* = 0.126, Kruskal–Wallis) compared to the control. The opposite occurred at the end of this experiment (72 h), where the settling inhibitory activity was higher (49%) at 0.3% (*p* = 0.000, Kruskal–Wallis). Although fewer aphids were settled at concentrations of 0.1% (SI 11%) compared to the control, the difference was not statistically significant (*p* = 0.056, Kruskal–Wallis).

**Figure 3:**
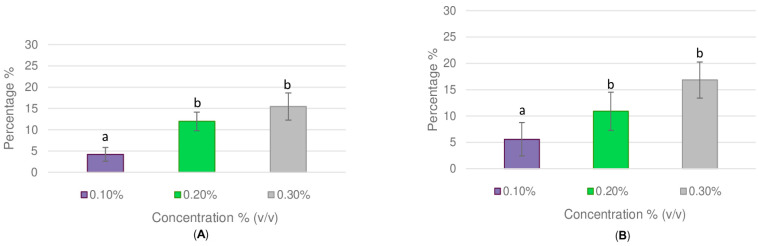
Mortality of chrysanthemum aphids caused by different concentrations of *C. capitatus* essential oil, as determined by (**A**) the dual-choice assay and (**B**) the no-choice assay. Results are expressed as mean values and standard errors for 20 biological replicates, with 10 adult aphids per treatment. Significance was set at *p* ≤ 0.05 using the LSD test. Different letters indicate significant differences between mean values.

The impact of *C. capitatus* essential oil at concentrations of 0.1% *v*/*v*, 0.2% *v*/*v*, and 0.3% *v*/*v* on the mortality percentage of chrysanthemum aphids after 72 h of treatment is shown in Figure 3. In general, the mortality increased in a dose-dependent manner as observed both by dual-choice (Figure 3A) and no-choice (Figure 3B) assays (ANOVA, *p* = 0.000). At the lowest concentration used (0.1% *v*/*v*), there was no significant effect on aphid mortality compared to the control (LSD, *p* = 0.130 for dual-choice assay, *p* = 0.117 for no-choice assay). The highest mortality rate, reaching 15% (*p* = 0.000, LSD) and 17% (*p* = 0.000, LSD), was observed after 72 h of exposure, according to the data of the dual-choice and no-choice test, respectively. Furthermore, it is important to mention that aphids exhibited neurotoxic symptoms, such as convulsions and tremors, particularly at the beginning (30 min) of the no-choice test using the highest concentration of essential oil (0.3% *v*/*v*). None of these symptoms was apparent at the lowest concentration (0.1% *v*/*v*) employed in the experiments.

### 2.2. Total Phenolic Content and Radical Scavenging Activity of the Extracts

The results of the total phenolic content of *C. capitatus* methanol extract, as determined using the Folin–Ciocalteu assay, and the antioxidant activities assessed using the DDPH method for the methanol extract and the essential oil are shown in Table 1. The mean value of the four replicates used to determine the total phenolic content in the methanol extract was 13.24 ± 0.17 mg of Gallic acid equivalence (GAE)/g dry weight (dw). The radical scavenging capacity of the extract, expressed as half the maximal inhibitory concentration (IC_50_) value, was 285.82 ± 3.49 μg/mL, and this was the same for the essential oil at 217.75 ± 5.45 μg/mL. The IC_50_ value of ascorbic acid used as an antioxidant compound for comparison in this assay was 4.42 ± 0.09 μg/mL.

### 2.3. Chemical Composition of Essential Oil

The chemical composition of *C. catipatus* essential oil used in the experiment was determined using Gas Chromatography–Mass Spectrometry (GC-MS). A total of 37 compounds were identified (Table 2) (Figure S1). The main compound of the essential oil was the oxygenated monoterpenic phenol Carvacrol (83.68%); in addition, important amounts of p-Cymene (6.30%) and γ-Terpinene (1.14%), which are biosynthetic precursors of Carvacrol and Thymol, were also present. The sesquiterpenic hydrocarbon fraction comprises a small part of the essential oil, with caryophyllene (1.33%) as the major constituent. Caryophyllene oxide was identified at a percentage of 1.27%. The remaining 32 components comprised a small portion (6.28%) of the essential oil.

### 2.4. Profile of Phenolic Compounds

Chemical analysis of the methanol extract of *C. capitatus* revealed the presence of fifteen (15) phenolic compounds (Table 3), (Figure S2). Five of them belong to the class of phenolic acids (Vanillic acid, Salicylic acid/4-hydroxybenzoic acid, Caffeic acid, Rosmarinic acid, and Salvianic acid A). Four were classified as flavones (Luteolin, Diosmetin, Apigenin, and Acacetin) and one was classified as Flavone C-Glycosides (Apigenin 8-C-glucoside). Other compounds included Naringenin (flavanone), Hesperidin (flavanone O-Glycoside), Taxifolin (flavanol), Quercetin (flavonol), and Salvianolic acid J (polyphenol).

## 3. Discussion

In recent years, there has been a growing interest in using essential oils derived from aromatic and medicinal plants for plant protection instead of synthetic pesticides. In addition to their low toxicity to mammals and non-target organisms, essential oils are characterized by their non-bioaccumulative nature in the environment, low risk of developing pest resistance, and beneficial effects on human health.

This study investigated the effects of *C. capitatus* essential oil on the behaviour and survival of *M. sanborni*, a monophagous aphid species that feeds on chrysanthemum plants. Moreover, it is responsible for virus transmission. Chemical analysis of the essential oil showed the presence of compounds that can affect aphid behaviour, while the antioxidant properties of the aromatic plant were tested to show its additional beneficial effects on health. In the experiments, the settling inhibitory activity and lethal effect of the tested essential oil were evaluated after exposing *M. sanborni* adults to treated leaf discs using dual-choice and no-choice tests. The results from the dual-choice test show that when provided with a different food option, aphids preferred to settle on untreated-control leaf discs over those with essential oil, and the extent of prevention of settlement was determined by both concentration and mortality rates. Τhe essential oil, for two hours after treatment, maintained its strong deterrent activity, which was gradually reduced thereafter, at all concentrations used. At the concentration of 0.3%, the prevention of aphid settlement still showed a considerable impact (46%) after 72 h, alongside a mortality rate of 15%. The initial strong deterrent effect of the essential oil, which diminishes over time, is possibly due to the volatilization of the bioactive components, leading to a limited lifespan on the leaf disc surface. Another possibility is that the aphid becomes accustomed to or insensitive to the substance that originally hindered its establishment.

In contrast to the dual-choice tests, under no-choice conditions, our experiments showed that the impact of essential oil on aphids settling on treated leaf discs varied over time, with a stronger inhibitory effect observed at the highest concentrations after 72 h, in contrast to the initial conditions of the experiment (30 min). This could be due to the toxic effect of the essential oil on chrysanthemum aphids, which was clear at higher essential oil concentrations. In particular, at the highest concentration (0.3%, *v*/*v*), symptoms like hyperactivity, convulsions, and tremors were more apparent. These symptoms are known [27] and compatible with the neurotoxic mode of action on chrysanthemum aphids. So, under no-choice conditions, the aphids initially attempted to settle on the leaf disc treated with the essential oil, but movement was delayed due to the toxic effect. The higher the concentration, the longer it took for the aphids to move from the treated leaf surface. This is why the settling inhibitory activity was higher after 30 min when a lower concentration was applied. A concentration of 0.3% *v*/*v* (highest) reduced aphid establishment by 49% within 72 h, with the number of dead aphids increased to 17%.

The adaptation of *C. capitatus* to different environmental conditions has led to the development of Carvacrol, Thymol, and intermediate Carvacrol/Thymol chemotypes. The plant material used for the experiments was collected from a wild population growing in a region of north Epirus, Greece. Chemical analysis of the essential oil using GC-MS revealed that it belonged to the Carvacrol chemotype. A high percentage of the phenolic compound Carvacrol (83.48%) has also been observed in Ikaria, a Greek island in Eastern Aegean [28]. The total phenolic content of the methanol extract is in agreement with the levels determined (13.15 ± 0.34 mg GAE/g of dw) for *C. capitatus* collected from different areas [29]. The LC-MS analysis led to the identification of sixteen phenolic compounds with established beneficial impact for human health. Phenolic compounds can scavenge and stabilize free radicals because of their hydroxyl units, either by donating hydrogen or electrons. Overproduction of free radicals may influence macromolecules such as DNA, proteins, and lipids, which may lead to serious health problems [30]. In food systems, it may also be a main parameter causing quality degradation. Both the essential oil and methanol extract of *C. capiatus* exhibited significant antioxidant activity. The essential oil required to cause 50% inhibition of the DPPH free radicals (217.75 ± 5.45 μg mL^−1^) was similar to that of *O. vulgare* ssp. *hirtum* essential oil (IC_50_ 220.59 ± 4.03 μg mL^−1^), which was analogous to Carvacrol (86.4%) [31]. The use of essential oils with phytoprotectant properties, especially those recovered from aromatic and medicinal herbs, is of interest because they are characterized as safe and are used as food additives. Such bioactive compounds (essential oils or plant extracts) can be added to biodegradable packaging products and incorporated into films/trays/nets, providing high antimicrobial and antioxidant capacity. As a result, the products have a longer shelf life, and the whole process will have less environmental impact. Regarding the toxicity of *C. capitatus* essential oil, researchers [32] have demonstrated its low oral acute toxicity in tests using mouse models (LD_50_ 2.000 mg Kg^−1^) and its lack of mutagenic activity [17,27]. Moreover, *C. capitatus* essential oil and its main components have insecticidal properties on *Drosophila melanogaster* [27] as well as the tulip aphid *Lipaphis pseudobrassicae* [33]. Carvacrol, the main component of *C. capitatus* essential oil, contributes not only to oregano’s distinct aroma but also has antioxidant, anti-inflammatory, anticancer, antimicrobial, and antifungal properties. Additionally, it has insecticidal or repellent effects on certain plant insect pests [34]. Several mechanisms could be involved in the manifestation of the neurotoxic activity of essential oil on *M. sanborni*. Some components, such as Carvacrol, can act as acetylcholinesterase inhibitors or affect GABA-gated dependent chloride channels [35]. Moreover, they can serve as ligands specific to invertebrate octopamine and tyramine receptors (OARs/TARs), affecting the physiological and cellular responses of insects [36]. Essential oils exhibit selective toxicity towards insects but not towards mammals, possibly because vertebrates lack octopamine receptors. Another component of *C. capitatus* essential oil, Linalool, has been shown to have insecticidal and repellent effects on *Aphis gossypii* [37]. Similar repellent activity was observed for the oxygenated monoterpenes α-Terpineol and 1,8-Cineole, against *M. persicae* and *Diuraphis noxia* [38]. In summary, the overall bioactivity of an essential oil results from the synergistic or antagonistic interplay of its ingredients. Unlike pure compounds, essential oils have the ability to target multiple sites of action simultaneously. However, the side effects of essential oils used as aphidicides or aphid repellents should be considered, particularly in relation to natural enemies, pollinators, and their possible phytotoxic activities.

## 4. Materials and Methods

### 4.1. Plant Material

The aerial parts of *Coridothymus capitatus* were collected during the flowering period, from wild populations on the mountain Tsouka Podogora-Zarkorache, municipality Ziros, Epirus, Greece. The selected plant material was dried in the shade. Voucher specimens were authenticated and stored in the herbarium at the OPENSCREEN-GR facility located at the University of Ioannina in Greece.

### 4.2. Essential Oil Preparation

Dry plant material (20 g) was chopped and subjected to hydrodistillation for two hours to recover the essential oil, using a Clevenger apparatus (manufactured in Wertheim, Germany). The essential oil, dried over anhydrous sodium sulphate (Na_2_SO_4_, Merck, Schnelldorf, Germany), was stored in a sealed vial at 4 °C, under N_2_, until use.

### 4.3. Preparation of Methanol Extracts

Methanol (MeOH, Sigma-Aldrich, Steinheim, Germany) extracts of powdered dry plant material (0.5 g dw/5 mL) were prepared via sonication using an ultrasonic bath (Bandelin Electronic, Berlin, Germany) (3 cycles × 5 min). The plant material was separated from the extract by centrifugation and stored at −20 °C until use.

### 4.4. Insect Culture

Chrysanthemum aphids, such as *M. sanborni,* were obtained from an area free of pesticide treatment for at least 5 years, located on the campus of the Agricultural Department, University of Ioannina in Arta, Epirus, Greece. A colony of 10 apterus female individuals was established on chrysanthemum plants. *M. sanborni* were reared in an insectarium at 22 ± 2 °C, relative humidity 70 ± 5%, and a photoperiod of 16:8 L:D. Bioassays were conducted under the same conditions.

### 4.5. Bioassays

The effect of the essential oil on aphid behaviour and survival was studied using dual-choice and non-choice bioassays. Three different essential oil concentrations (0.1%, 0.2%, and 0.3%) were used for the experiments. For the preparation, an appropriate volume of the essential oil was dissolved in Triton X-100 (Merck, Schnelldorf, Germany) and made up to 100 mL with distilled water. All the essential oil emulsions contained 0.5% Triton X-100. 

A Petri dish (9 cm diameter) was used as a test chamber for the experiments. Two sheets of filter paper were placed at the bottom and moistened with 2 mL of distilled water. For all experiments, a standard chrysanthemum leaf disc (2 cm diameter), immersed in the essential oil emulsion for 2 s, was used. Control chrysanthemum leaf discs were immersed in 0.5% Triton X-100 in the absence of essential oil.

#### 4.5.1. Dual-Choice Test

A dual-choice bioassay was conducted to evaluate the effect of *C. capitatus* essential oil on apterous adult chrysanthemum aphids at 22 °C. An essential oil-treated and a control-untreated chrysanthemum leaf disc were placed in a Petri dish on top of the filter paper at approximately 1 cm distance. Ten adults of the same age from the laboratory culture were collected using a soft, fine brush and placed in an empty Petri dish until the beginning of the experiment. Under these conditions, the aphids were allowed to choose between the treated and the control leaf discs. Each treatment was replicated forty (40) times. The number of aphids on the treated and control leaf disc was counted after 30 min, 60 min, 120 min, 24 h, 48 h, and 72 h of exposure. A settling inhibition index (%SI) was calculated for each tested concentration as follows [39]:

% SI = 1 − [(%T/%C)] × 100

where %T is the percentage of aphids on the treated surface; %C is the percentage of aphids on the control surface.

Mortality was confirmed by touching the aphids with a fine brush.

#### 4.5.2. No-Choice Test

This assay was performed using a similar procedure to the dual-choice test, with the difference that only one leaf disc (control or treated with the essential oil) was placed in the centre of the experimental chamber. Each treatment was replicated twenty (20) times. The evaluation method was the same as described above.

### 4.6. Free Radical Scavenging Capacity

The free radical scavenging activity (RSA) of the samples was determined according to Yfanti et al. [31]. In brief, methanolic solutions of 0.5 to 4 mg mL^−1^ from the extract and 0.5 to 3 mg mL^−1^ from the essential oil concentrations were prepared. Afterwards, 100 μL of each solution was introduced into 900 μL of methanolic solution DPPH (Sigma-Aldrich, Steinheim, Germany), (with a 0.1 mM final concentration in DPPH), and the absorbance at 517 nm was measured after 30 min staying in the dark. The results were calculated in response to a standard curve of ascorbic acid. The percentage of scavenging activity was calculated using the following equation:

%DPPH scavenging activity = [(A_C_ − A_S_)/As] × 100

where As is the absorbance of the tested solution; A_C_ is the absorbance of the control.

The IC_50_ (concentration of the tested solution required to achieve 50% inhibition) of the DPPH free radical scavenging activity was calculated from a graph of percent inhibition (%) against the concentration.

### 4.7. Determination of the Total Phenolic Content

The total phenolic content of the tested solutions was estimated using the colorimetric Folin–Ciocalteu (FC) assay as mentioned in ref. [40]. In short, a glass tube was filled with 4.5 mL of H_2_O, followed by the addition of 100 μL of the extract and 100 μL of the reagent (Supelco, Bellefonte, PA, USA). The mixture was vigorously agitated for 1 min and an additional 3 min; then, 300 μL of saturated Na_2_CO_3_ (Merck, Darmstadt, Germany) solution was added. The mixture was left to stand in the dark for 2 h, and then the absorbance was read at 760 nm. A Gallic acid (GA, Sigma-Aldrich, Steinheim, Germany) standard curve (0.5 to 30.0 mg L^−1^) was prepared, and the phenolic content of the *C. capitatus* extract was expressed as mg GAE g^−1^ dry weight.

### 4.8. Phytochemical Analysis

#### 4.8.1. Gas Chromatography–Mass Spectrometry GC-MS Analysis

Essential oil analysis was performed using a gas chromatograph (GC, Simazu 2030, Simadzu, Kyoto, Japan) equipped with a capillary column (Mega 5-MS, 30 m × 0.25 mm × 0.25 μm), interfaced with a mass spectrometer (GCMS-QPSERIES, Simadzu, Kyoto, Japan), under a specific temperature programme [41]. Initially, the oven temperature was increased from 60 °C to 110 °C at a rate of 3 °C min^−1^; it was kept isothermal for 10 min, raised to 150 °C at a rate of 3 °C min^−1^, and finally increased to 280 °C at a rate of 30 °C min^−1^ and kept at the final temperature for 5 min. The injector and interface temperatures were set at 250 °C and 300 °C, respectively. The MS ion source temperature was set at 240 °C, the electron impact was 70 eV, and the spectrum ranged from 50 to 550 *m*/*z*. The volume of the injections was 1 μL of diluted samples (1:200 *v*/*v* in Hexane). An autosampler (AOC-20i/s Shimadzu, Kyoto, Japan) was used for the analysis. The compounds were identified through the comparison of mass spectra with the Nist library data (MS search 2.3), and the percentage (%) of each component in the essential oil was determined relative to the total compounds identified. The results were validated by comparing the retention indices (RIs) with those of n-alkanes (C8–C20), using data from the literature.

#### 4.8.2. LC-LTQ/Orbitrap HRMS Analysis

Ultra-high-performance liquid chromatography (UHPLC) coupled with an LTQ-FT/Orbitrap XL 2.5.5 SP1 mass spectrometer (Thermo Fisher Scientific, Bremen, Germany) was used to analyze the chemical composition of the phenolic compounds extracted by MeOH as previously described [31]. The system was equipped with an ESI source, an automatic sample flow pump, and an autosampler (Accela AS 2.1.1, Thermo Fisher Scientific, Bremen, Germany). Separation was performed on a reversed-phase Hypersil GOLD column (100 mm × 2.1 mm, 1.9 μm) from Thermo (Bremen, Germany). The mobile phase consisted of phase A (0.1% formic acid in LC-MS water) and phase B (LC-MS methanol). Water and MeOH (LC-MS grade) were purchased by Fisher Scientific (Leicester, UK) and fic acid (FA), 98–100% purity, was obtained from Merck (Darmstadt, Germany). The analysis duration was 25 min. The flow rate was set at 400 μL min^−1^; the injection volume was 10 μL; the tray temperature was set at 15 °C; and the oven temperature was set at 40 °C. The ESI source conditions were as follows: sheath gas flow rates at 55 arbitrary units (au), aux gas flow rate at 20 au, capillary temperature at 350 °C, and spray voltage of 2.7 kV. Using the MS/MS data-dependent mode, the Orbitrap analyzer captured full-scan mass spectra at high resolution by scanning the top 6 intense ions in the linear trap. A 35% normalized collision energy (NCE) was consistently used in the analysis (CID, collision-induced dissociation) to reveal the fragmentation pattern of the compounds. The molecular ion formation of the phenolic compounds was used to identify them, with their characteristic fragments compared to those in the NIST Mass Spectral Library 2020 or existing literature. The Xcalibur v.2.2 software (Thermo Electron, San Jose, CA, USA) was used to control the instruments and was also used for mass spectra processing.

### 4.9. Statistical Analysis

Statistical analysis was performed using the SPSS package, version 26. Wilcoxon’s matched-pairs signed rank test was used for the dual-choice test to assess significant differences between the control and treated groups. Data from the no-choice test were analyzed using the non-parametric Kruskal–Wallis test. Mortality data were corrected using Abbot’s formula [42], and the percentage values were subjected to analysis of variance (one-way ANOVA), followed by the least significant difference test (LSD) to compare means. Significance was set at *p* ≤ 0.05.

## 5. Conclusions

In conclusion, our findings indicate that the essential oil of *C. capitatus* affects the behaviour and survival of the chrysanthemum aphid *M. sanborni*. Under conditions where the aphid could choose its host, the essential oil prevented aphids from settling on the leaf discs more effectively than under no-choice conditions. The effectiveness of *C. capitatus* essential oil as a successful control agent depends on its ability to remain active on the leaf disc surface for a longer period, which can be optimized under a proper formulation. Symptoms consistent with a neurotoxic mode of action were more pronounced at higher concentrations. There was a marked increase in mortality rates at concentrations above 0.1%, *v*/*v*. The *C. capitatus* plant used in the present study is of the Carvacrol chemotype. Sixteen phenolic compounds were identified in the MeOH extract, and both the oil and MeOH extract exhibited significant antioxidant activity. Although the use of *C. capitatus* essential oil is considered safe for mammals as well as being eco-friendly, the impact on aphids’ predators and parasitoids must be explored.

## Figures and Tables

**Table 1:** Total phenolic content and radical scavenging activity.

Extracts/Standard	DPPH Assay IC_50_ (μg/mL)	Total Phenolic Contents (mg GAE/g dw)
Methanol extract	285.82 ± 3.49	13.24 ± 0.17
Essential oil	217.75 ± 5.45	
Ascorbic acid	4.42 ± 0.09	

**Table 2:** Chemical composition of *C. capitatus* essential oil.

S/N	RT	RI_Lit_	RI_Exp_	Compound Name	Area % *	Identification Method
1	5.864	926	926	α-Thujene	0.10	MS, RI
2	6.100	934	934	α-Pinene	0.30	MS, RI
3	6.617	951	951	Camphene	0.12	MS, RI
4	7.464	980	980	1-Octen-3-ol	0.24	MS, RI
5	7.732	989	989	Myrcene	0.73	MS, RI
6	8.030	999	999	3-Octanol	0.08	MS, RI
7	8.402	1009	1009	α-Phellandrene	0.08	MS, RI
8	8.450	1010	1010	δ-3-Carene	<0.05	MS, RI
9	8.760	1018	1018	α-Terpinene	0.56	MS, RI
10	9.075	1027	1027	p-Cymene	6.30	MS, RI
11	9.210	1030	1030	Limonene	0.18	MS, RI
12	9.306	1033	1033	β-Phellandrene	0.15	MS, RI
13	9.378	1035	1035	1,8-Cineole	<0.05	MS, RI
14	9.800	1046	1046	trans-β-Ocimene	<0.05	MS, RI
15	10.307	1059	1059	γ-Terpinene	1.14	MS, RI
16	10.846	1073	1073	cis-Sabinene hydrate	0.25	MS, RI
17	11.376	1087	1087	α-Terpinolen	0.06	MS, RI
18	11.657	1094	1094	p-Cymenene	<0.05	MS, RI
19	11.978	1102	1102	Linalool	0.65	MS, RI
20	12.113	1105	1105	trans-Sabinene hydrate	0.16	MS, RI
21	13.102	1128	1128	cis-p-Menth-2-ene-1-ol	<0.05	MS, RI
22	13.872	1146	1146	trans-p-Ment-2-en-1-ol	<0.05	MS, RI
23	15.198	1177	1177	Borneol	0.69	MS, RI
24	15.534	1184	1184	Terpinen-4-ol	0.54	MS, RI
25	15.892	1193	1193	p-Cymen-8-ol	<0.05	MS, RI
26	16.204	1200	1200	α-Terpineol	0.10	MS, RI
27	18.662	1254	1245	Carvone	0.10	MS, RI
28	21.221	1292	1292	Thymol	0.43	MS, RI
29	21.976	1303	1303	Carvacrol	83.68	MS, RI
30	31.170	1416	1416	β-Caryophyllene	1.33	MS, RI
31	33.651	1453	1453	α-Humulene	0.05	MS, RI
32	37.028	1506	1506	β-Bisabolene	0.25	MS, RI
33	40.441	1584	1584	Spathulenol	<0.05	MS, RI
34	40.586	1588	1588	Caryophyllene oxide	1.27	MS, RI
35	41.270	1610	1610	Humulene epoxide II	0.05	MS, RI
36	42.199	1679	1678	Germacra-4(15),5,10(14)-trien-1α-ol	0.12	MS
37	42.375	1691	1691	α-Bisabolol	<0.05	MS, RI
Oxygenated monoterpenes					86.73	
Sesquiterpene hydrocarbons					2.90	
Oxygenated sesquiterpenes					0.22	
Others					0.32	
Monoterpenes					86.73	
Sesquiterpenes					3.12	

* Percentage of each compound (%) over the total identified ones. **RT**: retention time; **RI_Lit_**: literature retention index; NIST *Chemistry Webbook*; SRD 69; **RI_Exp_**: experimentally determined retention index.

**Table 3:** Phenolic compounds of *C. capitatus* MeOH extract, as identified using LC-LTQ/Orbitrap HRMS.

Identified Compounds	Molecular Formula	ESI	Ion Form	Theoretical *m*/*z*	Mass Error (ppm)	MS/MS Fragments
Vanillic acid	C8H8O4	-	[Μ-H]^−^	167.0339	1.435	**123.13**/152.08/108.08
Salicylic acid/4-hydroxybenzoic acid	C7H6O3	-	[Μ-H]^−^	137.0244	1.669	**93.05**
Caffeic acid	C9H8O4	-	[Μ-H]^−^	179.0350	0.715	**135.09**
Salvianolic acid J	C27H22O12	-	[Μ-H]^−^	537.1038	0.638	**339.05**/493.11
Taxifolin	C15H12O7	-	[Μ-H]^−^	303.0499	1.446	**285.06**/177.12/125.02
Apigenin 8-C-glucoside	C21H20O10	-	[Μ-H]^−^	431.0984	0.807	**311.07**/341.14
Rosmarinic acid	C18H16O8	-	[Μ-H]^−^	359.0772	1.766	**161.03**/197.03/179.04
Salvianic acid A (Danshensu)	C9H10O5	-	[Μ-H]^−^	197.0445	1.220	**179.01**
Hesperidin	C28H34O15	-	[Μ-H]^−^	609.1814	0.063	**301.09**
Luteolin	C15H10O6	-	[Μ-H]^−^	285.0405	1.225	**241.09**/175.08/199.08
Quercetin	C15H10O7	-	[Μ-H]^−^	301.0354	−0.019	**179.01**/151.09
Diosmetin	C16H12O6	-	[Μ-H]^−^	299.0561	1.225	**284.09**/285.04
Apigenin	C15H10O5	-	[Μ-H]^−^	269.0455	2.120	**225.07**/149.02/201.10
Naringenin	C15H12O5	-	[Μ-H]^−^	271.0612	1.240	**151.00**/177.09
Acacetin	C16H12O5	-	[Μ-H]^−^	283.0601	2.000	**268.11**

Τhe most abundant MS/MS fragments are marked in bold.

## Data Availability

The authors declare that datasets will be made available on request.

## Supplementary Materials

The following supporting information can be downloaded at https://www.mdpi.com/article/10.3390/molecules30163437/s1. Figure S1: GC-MS chromatogram of *C. capitatus* essential oil; Figure S2: LTQ/Orbitrap HRMS chromatogram of *C. capitatus* methanol extract in negative ion mode.
