# Supplementary material for: Effect of Coridothymus capitatus Essential Oil on Chrysanthemum Aphid Behaviour and Survival: Phytochemical Analysis and Antioxidant Potential
Source: Molecules. 2025 Aug 20;30(16):3437. doi: 10.3390/molecules30163437 (PMC12388747; doi:10.3390/molecules30163437)
Supplement: Supplementary file 1 [file molecules-30-03437-s001.zip › Supplementary Figure S1.pdf]

Effect of *Coridothymus capitatus* essential oil on chrysanthemum aphid behaviour and survival. Phytochemical analysis and antioxidant potential.

Paraskevi Yfanti [1,*], Andreas Papavlasopoulos [3], Polyxeni Lazaridou [2], Douma Dimitra [3] and Marilena E. Lekka [2,*]

[1] Department of Agriculture, University of Ioannina, 47150 Arta, Greece; pyfanti@uoi.gr

[2] Department of Chemistry, University of Ioannina, 45110 Ioannina, Greece; p.lazaridou@uoi.gr, mlekka@uoi.gr

[3] Independent Researcher, 45332 Ioannina, Greece; andreas_papavlasopoulos@yahoo.com; dimitra@ddoumasons.com

* Correspondence: mlekka@uoi.gr (M.E.L.); pyfanti@uoi.gr (P.Y.); Tel.: +30-26510-08367 (M.E.L.); +30-26810-50248 (P.Y.)

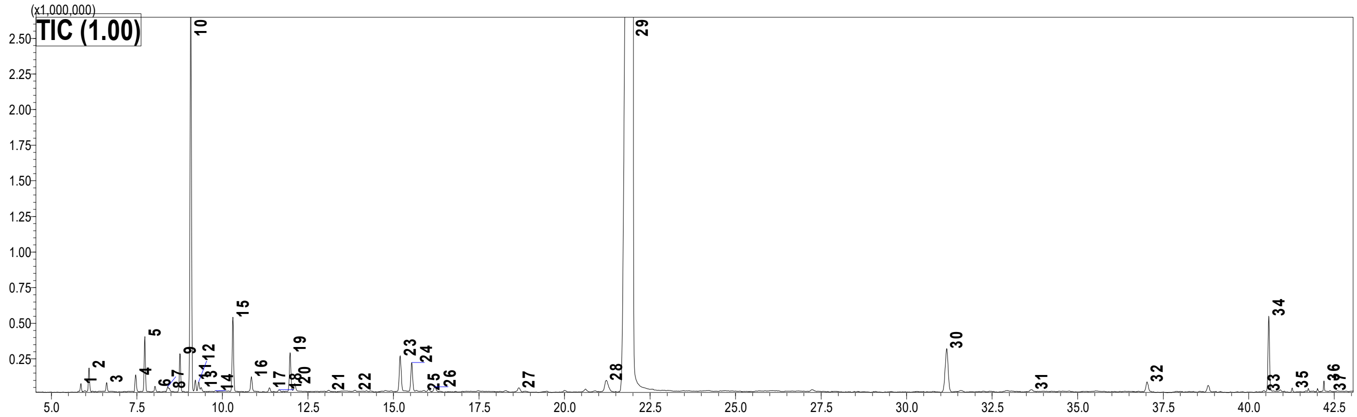

Figure S1: GC-MS chromatogram of *C. capitatus* essential oil
